# An empirical Bayes model using a competition score for metabolite identification in gas chromatography mass spectrometry

**DOI:** 10.1186/1471-2105-12-392

**Published:** 2011-10-10

**Authors:** Jaesik Jeong, Xue Shi, Xiang Zhang, Seongho Kim, Changyu Shen

**Affiliations:** 1 Department of Biostatistics, Indiana University, 410 West 10th Street, Indianapolis, IN 46202, USA; 2 Department of Chemistry, University of Louisville, 2320 South Brook Street, Louisville, KY 40292, USA; 3 Department of Bioinformatics and Biostatistics, University of Louisville, 485 E. Gray St, Louisville, KY 40292, USA

## Abstract

**Background:**

Mass spectrometry (MS) based metabolite profiling has been increasingly popular for scientific and biomedical studies, primarily due to recent technological development such as comprehensive two-dimensional gas chromatography time-of-flight mass spectrometry (GCxGC/TOF-MS). Nevertheless, the identifications of metabolites from complex samples are subject to errors. Statistical/computational approaches to improve the accuracy of the identifications and false positive estimate are in great need. We propose an empirical Bayes model which accounts for a competing score in addition to the similarity score to tackle this problem. The competition score characterizes the propensity of a candidate metabolite of being matched to some spectrum based on the metabolite's similarity score with other spectra in the library searched against. The competition score allows the model to properly assess the evidence on the presence/absence status of a metabolite based on whether or not the metabolite is matched to some sample spectrum.

**Results:**

With a mixture of metabolite standards, we demonstrated that our method has better identification accuracy than other four existing methods. Moreover, our method has reliable false discovery rate estimate. We also applied our method to the data collected from the plasma of a rat and identified some metabolites from the plasma under the control of false discovery rate.

**Conclusions:**

We developed an empirical Bayes model for metabolite identification and validated the method through a mixture of metabolite standards and rat plasma. The results show that our hierarchical model improves identification accuracy as compared with methods that do not structurally model the involved variables. The improvement in identification accuracy is likely to facilitate downstream analysis such as peak alignment and biomarker identification. Raw data and result matrices can be found at http://www.biostat.iupui.edu/~ChangyuShen/index.htm

**Trial Registration:**

2123938128573429

## Background

The metabolome represents the collection of small compound metabolites in an organism or biological system, typically under 1000 daltons [1]. The network of metabolic reactions, where outputs from one enzymatic chemical reaction are inputs to other chemical reactions, is a key component of the cellular physiology. In addition, the interactions of metabolites with other larger bio-molecules (i.e. proteins) are critical for many important biological processes. Therefore, metabolomics, the study of all metabolites in a system, in its own has great implications in scientific and biomedical advancement [2,3].

**Figure 1:**
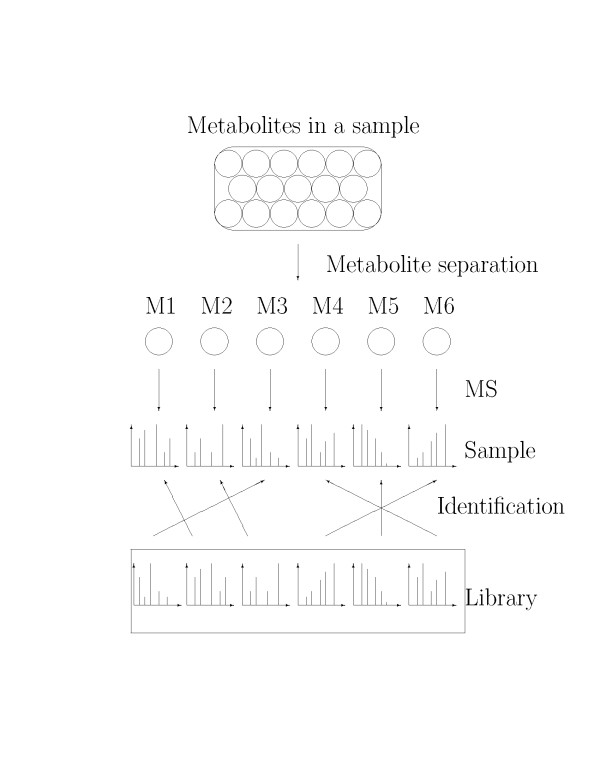
**Schematic representation of the GCxGC/TOF-MS experiment**. Schematic representation of the GCxGC/TOF-MS experiment. M1,...,M6 are metabolites in a sample after separation. "Library" includes spectra of the known identities.

Mass spectrometry is a popular technique for metabolic profiling [4]. In a typical experiment, metabolites in a sample are first derivatized and then separated using either liquid or gas chromatography (LC/GC). The separated metabolites are further analyzed by mass spectrometry to generate their fingerprint spectra (see Figure 1). The identification of a metabolite is usually assisted by including a spectrum library where each spectrum's identity is known. The fingerprint spectrum is compared with each spectrum in the library using a numerical score that characterizes the similarity of the pair. The score used to measure mass spectral similarity is called similarity score. The metabolite in the library with the best score is matched to the fingerprint spectrum as the identification of the spectrum [5] (see Figure 1).

With the development of mass spectrometry technology, particularly combined with the comprehensive two-dimensional gas chromatography (GCxGC) that substantially improves the separation capacity, a large number of metabolites can now be identified at a time. By comparing spectra from those metabolites with spectra from known identities, identification is performed [6]. However, these identifications are subject to errors due to experimental noise, incompleteness of the library, technical limitations and so on. Thus, it is in great need to improve the accuracy of both the identifications and estimates of false positives at the data analysis stage as the validity and efficiency of the downstream analyses rely on the quality of the identifications.

To our knowledge, there have been relatively few developments along this line, compared with similar analysis issues in mass spectrometry based proteomics [5,7-9]. Several studies on spectra registration (or alignment) for comprehensive two-dimensional GC data have been done [10-13]. In some studies, without addressing the identification issue, they assumed that metabolite identification by ChromaTOF software is correct instead and used those identification results directly for alignment. In addition, no model to analyse score distribution has been developed in order to improve the accuracy of metabolite identification. In this paper, we propose an empirical Bayes model which analyzes similarity score distribution to improve the accuracy of metabolite identifications and their confidence measures for GCxGC/TOF-MS data. The model orchestrates all information coming from each experiment step and produces confidence measure of identification in the form of posterior probability. The advantages of our method include (i) the posterior probability allows straightforward estimation of false discovery rate (FDR) [14] and serves as the confidence measure; (ii) metabolites in the library that are not matched to any spectrum are also assigned a confidence measure regarding their presence/absence status in the sample; (iii) integration of different sources of evidence may provide better identification accuracy. A major novelty of our method is the inclusion of a competition score (*b_j_*) for each metabolite *j *in the library, which is defined based on all spectra in the library. The competition score is a measure of the propensity of *j *being matched to some sample spectra through the resemblance of the spectrum of metabolite *j *with other spectra. As explained in details in Methods Section, the competition score provides useful information to discern the true from the false positives. In what follows, we provide a detailed description of the model and demonstrate the utility of our model through analysis of a mixture of metabolite standards and a real data set generated by GCxGC/TOF-MS. For terminological clarity, since our identification is the compound identification via database search, it is also known as putative annotated identification. For simplicity, we use the word "identification" throughout the article.

## Results

### Experiments

#### Experiment 1: Mixture of metabolite standards

A mixture of 35 amino acids, fatty acids and organic acids were prepared in pyridine and derivatized with 100 *μL *of N-Methyl-N-(Tert-Butyldimethylsilyl) trifluoroacetamide (MTBSTFA). All GCxGC/TOF-MS analyses were performed on a LECO Pegasus 4D time-of-flight mass spectrometer (TOF-MS). Then, acquired data were processed with a user defined data processing method. The LECO ChromaTOF software version 3.41 was used for instrument control and spectrum deconvolution. A total of 3286 sample spectra were obtained from this experiment.

#### Experiment 2: Rat plasma data

Metabolites were extracted from a 100*μL *rat plasma sample using 900*μL *of organic solvent mixture (methanol:water = 8:1) and further derivatized with MTBSTFA. All GCxGC/TOF-MS analysis were performed on a LECO Pegasus 4D time-of-flight mass spectrometer (TOF-MS). The acquired data were processed with a user defined data processing method. The same software, LECO ChromaTOF software version 3.41 was used for instrument control and spectrum deconvolution. 1122 sample spectra were obtained from the experiment.

More details about both experiments are provided in the Additional file Additional File 1.

### Library and database search

For the analysis of Experiment 1 data, because of the derivatization, a total of 52 metabolites are considered to be the true positives. Details of the 52 true positives are provided in the Additional file. The spectra of the 52 true positives were extracted from NIST/EPA/MIH Mass Spectral Library (NIST2002) that was embedded in ChromaTOF software. To introduce noises, we also randomly selected 2000 false spectra from the same library. Therefore, the library is composed of *N *= 2052 spectra with known identities. We consider cosine score as the similarity score for a pair of spectra. The cosine score (S) is interpreted as the angle between two spectra with smaller angle indicating more similarity, which is defined

(1) $$S=\frac{180}{\pi }cos^{-1}\left(\frac{<A,B>}{\left||A||\cdot ||B|\right|}\right),$$

where *< A*, *B >*is the inner product of spectra *A *and *B *and || · || is the Euclidean norm. To calculate the competition score *b_j_*, we conducted all pairwise comparisons among the 2052 spectra in the library and considered two values for the threshold *h*, *h *= 30 and *h *= 40 (see Methods Section for the definition of *b_j _*and *h*). For each of the 3286 sample spectra, we compared it with the 2052 spectra from the library using cosine score and selected the metabolite with the best score (i.e. smallest) as the assignment to that sample spectrum.

For the analysis of Experiment 2, we used the library obtained from Automated Mass Spectral Deconvolution and Identification System (AMDIS) software, which includes 3540 spectra. AMDIS is a software for GC-MS data interpretation from National Institute of Standards and Technology (NIST). Again, *b_j _*was calculated based on all pairwise comparison of spectra in the library and database search assigned the metabolite with the best cosine score to each sample spectrum.

### Mixture of metabolite standards

**Figure 2:**
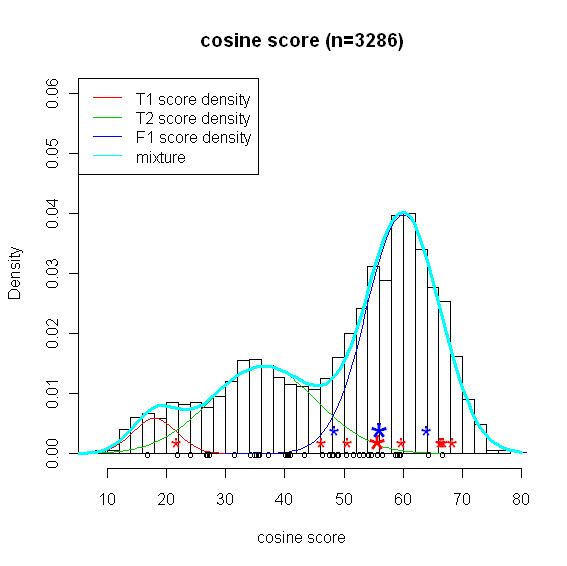
**Histogram of similarity scores and three estimated normal curves**. Mixture of metabolite standards: histogram of 3286 best scores obtained from searching the library and three estimated normal curves (red, green, blue) and mixture distribution (cyon). T1(red) and T2(green) are two components of the mixture density *f_T _*and F1(blue) is *f_F_*. The three component normal mixture for similarity score is the mixture of T1, T2, and F1. 43 true positives out of 52 are assigned to at least one sample spectrum and average score is calculated for metabolites matched more than one sample spectra. 43 average scores for the 43 true positives are indicated by black circle; For another example, we selected two metabolites with CAS number 107715-91-3 (TP1) and 6066826 (TN1). They have 8 and 2 match pairs, respectively. 8 scores for TP1 are indicated by red asterisks and 2 scores for TN1 are indicated by blue asterisks. Bigger asterisks (red, blue) indicate average of the scores with the same color.

Figure 2 is a histogram of the 3286 cosine scores from searching the library of 2052 spectra. Scores of the true positives are highlighted as circles at the bottom of Figure 2. Clearly, similarity scores of the true positives are in general better (i.e. smaller) than scores of the false positives. We consider a mixture model for the score distribution, which is composed of the distributions of true positive (*f_T_*) and false positive (*f_F_*). For our analysis, we consider *f_T _*to be composed of the two normal components on the left and *f_F _*to be the single normal component on the right. Here we present the results based on *h *= 30. Results based on *h *= 40 do not differ much and can be found in the Additional file.

**Figure 3:**
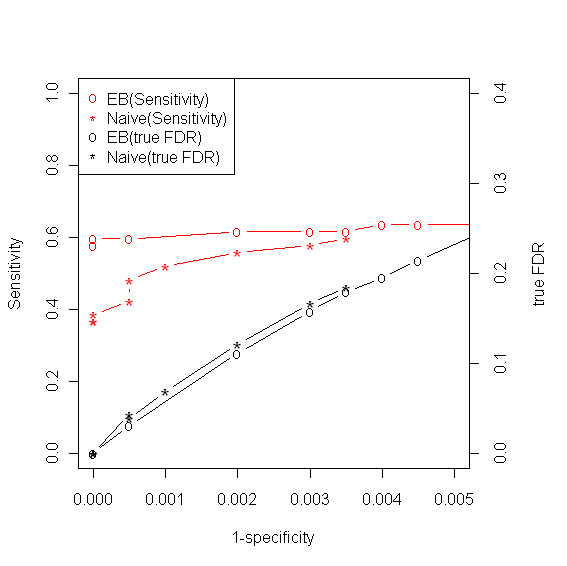
**ROC curves and FDR**. Mixture of metabolite standards: Receiver Operating Characteristic (ROC) curves and true FDR for empirical Bayes (EB) and naive methods.

We obtained $\hat{\rho }=0.026$ based on the EM algorithm. This suggests that about 2.6% of the metabolites in the library, or 2052 × 0.026 = 53, are expected to be present in our sample. This is very close to the known number of true positives, 52. Furthermore, $\hat{\tau }=0.84$, which indicates that 84% of the matches of the true positives are correct. The three fitted normal curves and the mixture distribution (three component normal mixture) are shown in Figure 2. It can be seen that the estimated mixture distribution (thick solid line in cyon) fits the similarity score data well (Goodness of fit test results are given in the Additional file). One major advantage of the mixture sample of metabolite standards is that it allows us to assess the performance of any method because the identities of metabolites in a sample are known. For comparison purpose, we consider four methods: naive method, NIST MS dot product, weighted dot product, and composite similarity based on the methods developed by Stein and Scott [15]. More details about three other methods except Naive method are given in [15,16]. Among them, the naive method has the best performance in terms of ROC curve and FDR. All comparison results are provided in the Additional file. Thus, we chose the naive method to compare with our method, which is defined by using the average similarity score to call the status of a metabolite (see Methods Section). Figure 3 shows the receiver operating characteristic (ROC) curves of our method and the naive method. In the same graph, we also plot the true FDR for both methods. It is clear that our method has more discriminating power than the naive method. Note that our library has much more true negatives than true positives, i.e. 2000 v.s. 52, implying that even minor compromise in specificity will significantly inflate the FDR. For instance, even though the specificity is controlled at 99%, the FDR is 28% with perfect sensitivity. Therefore, we chose to compare the ROC curve at high specificity range. As we can see in Figure 3, our method provides moderate sensitivity (60%) even at extremely high level of specificity (*>*99.9%).

**Figure 4:**
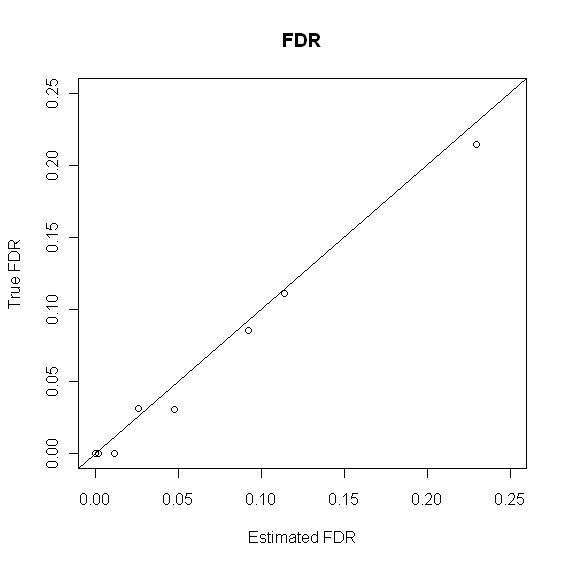
**True FDR v.s. Estimated FDR**. Mixture of metabolite standards: True FDR v.s. Estimated FDR.

Not only does our method provide better identification accuracy, but our method also provides a reliable estimate of the FDR. This is shown in Figure 4, where true FDR is plotted against estimated FDR for metabolites claimed to be present based on different thresholds of the posterior probabilities. It can be seen that the estimated FDR is very close to the true FDR and most of the time our FDR estimate is conservative.

The definitions of terminologies such as FDR, sensitivity, specificity and ROC curve are provided in the Additional file.

### Rat plasma

**Figure 5:**
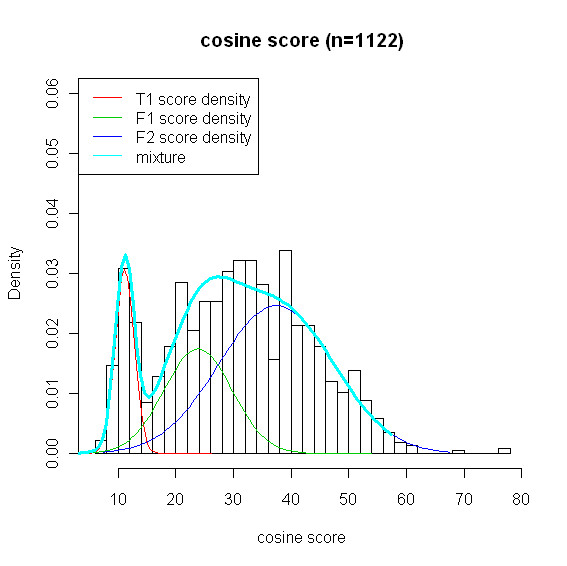
**Histogram of similarity scores and three estimated normal curves**. Rat plasma: histogram of 1122 best scores obtained from searching the library and three estimated normal curves (red, green, blue) and mixture distribution (cyon); T1(red) is *f_T_*, and F1(green) and F2(blue) are two components of the mixture density *f_F_*. The three component normal mixture for similarity score is the mixture of T1, F1, and F2.

We present the results based on *h *= 30. Results based on *h *= 40 can be found in the Additional file. Figure 5 shows the distribution of the similarity scores of the 1122 spectra generated by a sample of rat plasma. Similar to the experimental data of the mixture of metabolite standards, there seems to be three distribution components as well. We fit similar model as the one used for the analysis of the data of mixture of metabolite standards. As shown in Figure 5, the estimated normal mixture (thick solid line in cyon) fits the empirical data well (see Additional file for Goodness of fit plots).

Based on parameter estimates, we expect that 10% (or 354) of the 3540 metabolites in the library are present in the sample. In a perfect world, there should be 354 metabolites with posterior probability equal to 1 and the rest have zero posterior probability. If it is the case, we have the maximum amount of knowledge on which metabolites are present or absent. However, in the real world, the confidence is not concentrated on 354 metabolites, but diluted to all 3540 metabolites in the library. In Table 1 we provide the number of positives and nominal FDR by applying different thresholds to the posterior probabilities. Clearly, the probabilities are much more diluted to all 3540 metabolites in the library and only 79 of them have a confidence higher than 0.2. Therefore, our certainty on which metabolites are present or absent has been reduced. Essentially, the separation of the three distribution components is less obvious than the Experiment 1. As a result, discrimination of the true from the false metabolites in this experiment is more difficult. More results are given in the Additional file.

**Table 1:** Number of claimed positives and FDR

cutoff	NCP	EFDR	cutoff	NCP	EFDR
0.20	79	0.2873	0.25	73	0.2469
0.30	70	0.2265	0.35	67	0.2066
0.40	65	0.1933	0.45	63	0.1818
0.50	58	0.1522	0.55	55	0.1344
0.60	49	0.0988	0.65	46	0.0809
0.70	41	0.0524	0.75	40	0.0472
0.80	39	0.0420	0.85	35	0.0276
0.90	29	0.0098	0.95	28	0.0083

Number of claimed positives and FDR for Rat plasma. NCP stands for the number of claimed positives and EFDR stands for estimated false discovery rate

### Rationale behind the use of competition score and multiple similarity scores

In this section, we explain what kind of benefits we get from the use of competition score and multiple similarity scores of the same metabolite, the main advantages of our model. For this purpose, we present two illustrative examples by using Experiment 1. In the first example, we highlight the effective role of competition score in separating positives from negatives. To this end, we consider two hypothetical metabolites *j *= 1, 2, all of which are assigned to two sample spectra with the same similarity scores 40 and 50. However, the competition scores are different with (*b*_1_, *b*_1_*) = (0.1, 0.49), and (*b*_2_, *b*_2_*) = (1.08, 1.15), i.e., the only difference between two hypothetical metabolites is their competition scores. Then, any difference in the posterior probability is attributed to the difference in the competition score. Based on our model, the posterior probabilities for the two metabolites are 0.63 and 0.94, respectively. Therefore, the competition score allows adjustment of the confidence of a metabolite in addition to the similarity scores. To understand the difference in posterior probabilities, note that given the same similarity scores, the confidence of a metabolite is driven by the likelihood ratio of equation (4) to equation (3) as a function of *b_j _*and b_j_*. Metabolite 2 has higher confidence than metabolite 1 because the relative increase in likelihood of being matched due to the presence of metabolite 2 is higher than that of metabolite 1. To summarize, the idea is that evidence on the presence or absence status of a metabolite based on the fact that it is matched to some sample spectrum should be quantified according to the capability of the metabolite's spectrum to mimic other spectra in the library, and how the likelihood of being matched will be altered from absence to presence.

In the second example, we elucidate the treatment of our model on multiple similarity scores. More precisely, we highlight the effect of multiple similarity scores on confidence of metabolite identification. What if we use single average score of those multiple scores instead of individual scores? To answer this question, we select two metabolites with *b_j _*= 0 from the mixture of metabolite standards to exclude the effect of competition score: one true positive TP1 (CAS number:107715-91-3) and one true negative TN1 (CAS number: 6066826). Since we know which metabolite exists in sample in this case, a true positive presents a metabolite in library which exists in sample as well and is claimed as positive. TP1 was assigned to 8 sample spectra with similarity scores highlighted by * in red in Figure 3. TN1 was assigned to 2 sample spectra highlighted by * in blue. The bigger * represents the average score for each of the metabolites. The naive method is not able to discriminate the two metabolites because they have similar average scores, i.e. close to 56. However, our empirical Bayes model provides posterior probabilities 0.99 and 0.01 for TP1 and TN1, respectively. The reason our model performs well here is that all individual scores, not the single average score are incorporated into the model and play a key role of producing posterior probability. The point is that taking into account the number of matches and the distribution of the similarity scores provides rich information in discriminating the true from the false positives.

## Discussion

In this paper, there are two elements in layers 2 and 4 that need more explanation. The detail description of the model is given in Methods Section. In layer 2, the quadratic form of the function of conditional probability was inspired by the results of logistic regression. More specifically, we investigated the relationship between competition score and status of metabolites. For example, we fitted logistic regression model (linear and quadratic) to 2000 true negatives and noticed that quadratic function is fitted well, i.e., p-values for parameters corresponding to *b_j _*and *b_j_*^2 ^were less than 0.0001. In layer 4, the characterization of score function was based on the distributional behavior of similarity scores. It varies from data to data. In the data we analyzed, we considered three component normal mixture and the model worked reasonably well. Note that data-specific property of score distribution made us utilise different component mixing for each data set. For the mixture of standard metabolites, we considered *f_T _*to be two component normal mixture and *f_F _*single normal distribution. In contrast, for the rat plasma data, we considered *f_F _*to be two component normal mixture and *f_T _*single normal distribution.

The composition of the library will influence identification process. Obviously the quality and species of the spectra included in the library will affect how well identification can be made. However, this aspect is complicated and beyond of the scope of this paper. Actually it can be a separate study in its own right. Nevertheless, we did make observations on the effect of the size of the library on the quality of identifications. When the library include much more false positives, identification of component in the standard mixtures become much more difficult as there are "false positives" with great similarity with the true positives. The posterior probability in this case tend to be much lower. Note that this is not a failure of our model itself, it is simply because the matching score is not sufficiently discriminating anymore.

## Conclusion

Database-search based algorithm has been a popular approach to mass spectrometry-based high-throughput metabolite profiling. Due to the complexity of the experimental procedure and dynamic nature of the fragmentation process, identifications of metabolites in GCxGC/TOF-MS are subject to errors. The accuracy of identifications and false positive estimate are critical for the error control of downstream in silico or experimental investigations. During the database search, each candidate metabolite faces competition from other metabolites in the library to be the top hit. On the other hand, a large number of sample spectra also offer many opportunities for a candidate metabolite to be falsely matched. Taking into account the competition and opportunity associated with each candidate metabolite allows one to more properly extract evidence on the presence/absence status based on whether or not a candidate metabolite is matched to some sample spectra. This type of evidence adds another dimension of information to the similarity score for the assessment of the confidence of metabolite identifications. In this article, we proposed the concept of a competition score to characterize the magnitude of competition and opportunity for each candidate metabolite. The competition score and similarity score are integrated by an empirical Bayes model to yield confidence measure in the form of posterior probability.

Since our method is a novel model-based approach to metabolite identification in GCxGC/TOF-MS data, there is no other model-based method to compare with. Thus, we compared it with four other methods, especially, the naive method which is solely based on the similarity score. Through the experiment of mixture of metabolite standards, it was demonstrated that our model provides more accurate metabolite identifications than other methods. Just as controlling type I error is very important issue in classical statistics problem, so is controlling false discovery rate in high-throughput data [14,17,18]. As we see in Figure 4, our estimate of FDR is reliable and conservative. From a sensitivity perspective, a moderate sensitivity (about 0.6) is retained even at the extremely high level of specificity (greater than 0.999). It should be noted that the primary goal of high-throughput data analysis is to select promising candidate for downstream target-orientated experimental studies. Therefore, false positive is more of the investigator's concern than false negative. In this sense, we consider FDR is of higher priority than sensitivity in data analysis.

It is conceivable that the conditional probability of being a correct match given the presence status (i.e. *Pr*[*W_jl _*= 1|*Y_j _*= 1, *Z_j _*= 1]) may also depend on the competition score or some other more appropriate measures. Extension of our model along this direction could lead to further accuracy improvement.

## Methods

### Overview

**Figure 6:**
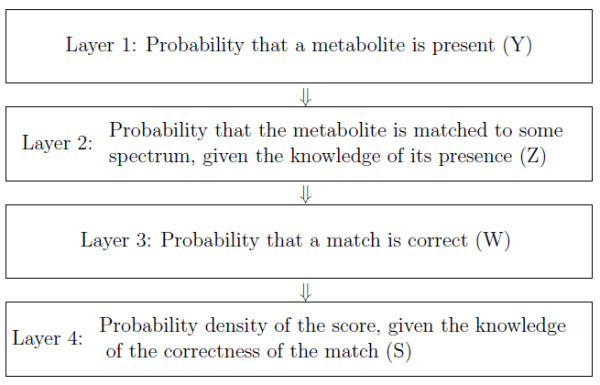
**Schematic representation of the hierarchy of the model**. Schematic representation of the hierarchy of the model (*Z *and *S*: observed, *Y *and *W*: unobserved).

The empirical Bayes model for metabolite identifications shares a similar structural hierarchy as the model constructed for peptide/protein identifications in [9]. Essentially, our model includes four layers that characterize the process through which the data are generated. The four layers target on four key variables relevant to metabolite identifications, which are the presence/absence of a metabolite in the sample (*Y*), whether or not a metabolite is matched to any sample spectrum (*Z*), whether or not a match is correct (*W*) and the similarity score (*S*). The rationale behind the model is that the information embedded in *Z *and *S *(observed) provides us evidence on the values of *Y *and *W *(unobserved). By properly establishing the connections between the two sets of variables, we can infer *Y *and *W *statistically. Hence, the empirical Bayes model partitions the joint distribution of the four variables into a series of marginal/conditional distributions starting with the marginal distribution of *Y *(see Equation 6). Specifically, the introduction of *W *allows correlations among multiple similarity scores of the same metabolite and improves identification accuracy [9]. Figure 6 presents a schematic representation of the hierarchical model. To describe our model, we adopt the following notations. For each metabolite *j *in the library, *Y_j _*= 1 or 0 indicates the true status of metabolite *j *with regard to its presence or absence in a sample; *Z_j _*= 1 or 0 indicates whether or not metabolite *j *is matched to some sample spectrum; For metabolite *j *matched to more than one sample spectra, *W_jl _*= 1 or 0 indicates whether or not the match of metabolite *j *to *l*th assigned sample spectrum is correct and *S_jl _*is the corresponding similarity score.

### Other identification methods

Since there is no other model-based approach other than our method, we compare our method with four other identification methods: naive method, NIST MS dot product, weighted dot product, and composite similarity based on the methods developed by Stein and Scott [15]. Among the four methods, the naive method has best performance in terms of ROC curve and FDR. Comparison results are provided in the Additional file. Thus, for comparison, we focus on the naive method, which is defined by using the same similarity scores which is incorporated in our model-based method. As mentioned, the similarity score is calculated by comparing library and sample spectra. Given the similarity score, the process of the naive method consists of three steps. First, we find the best match of each sample spectrum, i.e. a library spectrum with the best score obtained by the comparison of sample and library. Second, for those metabolites matched to more than one sample spectrum, we take average of the scores. Third, after applying cutoff value to the average score, we claim each metabolite as either positive or negative. On the other hand, metabolites in the library not matched to any sample spectrum are considered as absent in the sample.

### The model

**Layer 1: **We consider the marginal probability that each metabolite in the spectrum library is present in a sample:

(2) $$P\left(Y_{j}=1\right)=\rho ,\;j=1,2,\cdots \;,N,$$

where *N *is the number of the spectra in the library.

**Figure 7:**
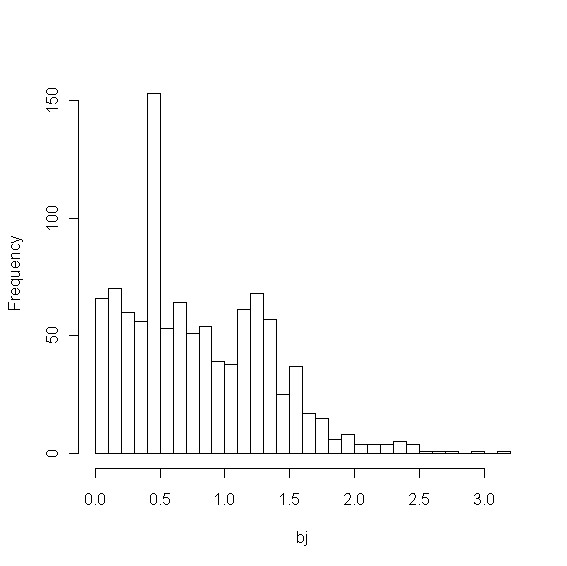
**Histogram of competition scores**. Histogram of competition scores greater than 0, i.e., *b_j _>*0, for the library searched against in Experiment 1.

**Layer 2: ***Z *is the first piece of information that provides us knowledge on the value of unobserved *Y*. For a given library, each metabolite has its own intrinsic intendancy to be matched to some sample spectrum due to the nature of the metabolite itself and the library. We need to account for this factor when we assess the confidence in *Y *using *Z*, i.e., magnitude of confidence in *Y *according to *Z*. To illustrate this idea, we consider the following hypothetical example. Suppose the spectrum of metabolite M1 shares high level of similarity with a large number of other metabolites. Then there is a high probability that it will be mistakenly matched to some sample spectrum of other metabolites even if M1 is absent in the sample (*Z*_*M*1 _= 0). What if M1 exists in the sample? In this case, M1's presence in the sample (*Z*_*M*1 _= 1) does not add much probability of being matched to some spectrum because there is high likelihood of being matched already regardless of the status of M1. Therefore, there is not much information in *Z*_*M*1 _on the value of *Y*_*M*1_. On the other hand, if metabolite M2 has a very unique spectrum, then its chance of being mistakenly matched to some spectrum generated by other metabolites is very low, and the presence of M2 in the sample increases the chance of being matched substantially. In this case, *Z*_*M*2 _provides us a lot of information on the value of *Y*_*M*2_. Therefore, proper assessment of the confidence in *Y *based on *Z *needs to be tailored to each individual metabolite. Along this line, we introduce a competition score, *b_j_*, for each metabolite *j *in the library. The *b_j _*is defined using the metabolite library in the following way:

$$b_{j}=\underset{{}_{k\neq j,k\in C,I\left(r_{kj}<h\right)}}{\sum }1∕a_{k},$$

where $a_{k}=\sum_{q\in C}I\left(r_{qk}<h\right)$, *r_qk _*is a similarity score between spectra of metabolites *q *and *k *in the library and it is assumed that the smaller the similarity score, the more resemblance the two spectra are (see Results Section for details of the definition of similarity score), *C *is the set of spectra in the library, and *I*(·) is the indicator function. In other words, *a_k _*is the number of neighbors of metabolite *k *within the radius of *h *including *k *itself (*a_k _*= 1, 2, 3, ⋯). Each neighbor is assigned "equal chance" of being matched to metabolite *k*. Therefore, the competition score *b_j _*is the characterization of the sum of the "chance" of metabolite *j *being matched to a spectrum generated by any of *j*'s neighbors (*k *is a neighbor of *j*). If metabolite *j *does not have any radius-*h *neighbor, then *b_j _*= 0. For the library used in our analysis of the mixture of metabolite standards, about 50% of the metabolites in the library has *b_j _*= 0 if *h *is set to 30. Figure 7 shows the histogram for those with *b_j _>*0. Intuitively, *b_j _*characterizes the propensity of *Z_j _*= 1 when *Y_j _*= 0 because the definition of *b_j _*does not include 1*/a_j_*. However, it should be noted that the effect of *b_j _*on *Z_j _*might not be monotone. This point is supported by a logistic regression of *Z_j _*for those *j *with *Y_j _*= 0 using a mixture of metabolite standards. The model demonstrated that there is high statistical significance that the log odd of *Z_j _*is associated with *b_j _*(for those metabolites with *b_j _>*0) through a downward quadratic curve, which results in the characterization of *γ*(*β*; *b_j_*). In consideration of the relatively large percentage of metabolites with *b_j _*= 0, we fit the following model for *Z_j _*conditional on *Y_j _*= 0, i.e., mixture of point mass and *γ(β*; *b_j_*)

(3) $$P\left[Z_{j}=1|Y_{j}=0\right]={\eta_{0}}^{I\left(b_{j}=0\right)}\gamma {\left(\beta ;b_{j}\right)}^{I\left(b_{j}>0\right)},$$

where $\gamma \left(\beta ;b_{j}\right)=1-\frac{1}{1+\exp\left(\beta_{0}+\beta_{1}b_{j}+\beta_{2}{b_{j}}^{2}\right)}$.

We consider the following competition score to characterize the propensity of being matched to some spectrum when *Y_j _*= 1:

$$b_{j}^{*}=\underset{k\in C,I\left(r_{kj}<h\right)}{\sum }1∕a_{k}.$$

In contrast to *b_j_*, b_j_* includes metabolite *j *itself as a neighbor to account for the fact that *Y_j _*= 1. The propensity of being matched should then be increased, which is reflected by the relationship b_j_* *> b_j_*. Note that *b_j _*= 0 indicates b_j_* = 1. We then fit the following model for *Z_j _*conditional on *Y_j _*= 1

(4) $$P\left[Z_{j}=1|Y_{j}=1\right]={\eta_{1}}^{I\left({b_{j}}^{*}=1\right)}\lambda {\left(\alpha ;{b_{j}}^{*}\right)}^{I\left({b_{j}}^{*}>1\right)},$$

where $\lambda \left(\alpha ;{b_{j}}^{*}\right)=1-\frac{1}{1+\exp\left(\alpha_{0}+\alpha_{1}{b_{j}}^{*}+\alpha_{2}{b_{j}}^{*2}\right)}$.

**Layer 3: **For those metabolites matched to at least one sample spectrum (*Z_j _*= 1), we consider the correctness of those matches. Obviously, if *Y_j _*= 0, the match is incorrect. For those matches of metabolite *j *with *Y_j _*= 1, we consider the following model:

(5) $$P\left(W_{jl}=1|Y_{j}=1,Z_{j}=1\right)=\tau ,$$

Hence, we assume that conditional on *Y_j _*= 1 and *Z_j _*= 1, the correctness of each assignment of *j *to some sample spectrum follows independent Bernoulli distribution. This layer allows our model to account for the situations where although a metabolite *j *is present in a sample, its assignment to some spectrum is not always correct. In other words, the match of a true positive to a sample spectrum may not always be correct.

**Layer 4: **we use a mixture model to characterize the distribution of the similarity scores.

$$f\left(S_{j}|W_{j}\right)=\underset{l}{\prod }f_{T}{\left(S_{jl};\phi_{T}\right)}^{W_{jl}}f_{F}{\left(S_{jl};\phi_{F}\right)}^{\left(1-W_{jl}\right)},$$

where *f *is the mixture of *f_T _*and *f_F _*that are the probability density functions of the scores of the correct matches and incorrect matches, respectively, and *ϕ_T _*and *ϕ_F _*are corresponding parameters.

By integrating the four layers, the joint distribution of variables involved in the model can be written as:

(6) $$\begin{array}{llll} \left[Y,Z,W,S\right] & =\left[Y\right]\left[Z|Y\right]\left[W|Y,Z\right]\left[S|W\right]\; & & \;\\ & =\left(\underset{j}{\prod }\left[Y_{j}\right]\right)\left(\underset{j}{\prod }\left[Z_{j}|Y_{j}\right]\right)\; & & \;\\ & \;\times \left(\underset{j:Z_{j}=1}{\prod }\underset{l}{\prod }\left[W_{jl}|Z_{j},Y_{j}\right]\;\left[S_{jl}|W_{jl}\right]\right)\; & & \;\\ & \; & \end{array}$$

By treating *Y *and *W *as the unobserved variables, we used Expectation-Maximization (EM) algorithm to estimate model parameters *θ *= (*ρ*, *η*_0_, *β*, *η*_1_, *α*, *τ*, *ϕ_T_*, *ϕ_F_*) [19]. The confidence of each metabolite *j *can then be calculated as the posterior probability of *Y_j_*:

$$P_{j}=\left\{\begin{array}{c} P\left[Y_{j}=1|Z_{j}=1,S_{j};\hat{\theta }\right]\\ P\left[Y_{j}=1|Z_{j}=0;\hat{\theta }\right] \end{array}\right)$$

where $\hat{\theta }$ is the estimated parameter vector. More details about EM are provided in the Additional file. It should also be noted that the posterior probability allows easy estimation of the false discovery rate (FDR). Specifically, the local false discovery rate (lFDR) in the sense of [20] can be estimated as

$$lFDR\left(j\right)=1-P_{j}.$$

The FDR for a set *Q *of *n *metabolites that is claimed to be present in the sample is [21]

$$FDR\left(Q\right)=\underset{j\in Q}{\sum }lFDR\left(j\right)∕n.$$

## Authors' contributions

JJ and CS designed and formulated the statistical model. JJ developed the programs to implement the model. XZ and XS designed the two experiments. XS conducted the experiments. SK conducted comparison study. All authors read and approved the final manuscript.

## Supplementary Material

Additional file 1 **File name: metabolomics-BMC bio-support**. This file include formula derivation and some results including tables and plots. Click here for file
