# Supplementary material for: An empirical Bayes model using a competition score for metabolite identification in gas chromatography mass spectrometry
Source: BMC Bioinformatics. 2011 Oct 10;12:392. doi: 10.1186/1471-2105-12-392 (PMC3228553; doi:10.1186/1471-2105-12-392)
Supplement: Additional file 1 — File name: metabolomics-BMC bio-support. This file include formula derivation and some results including tables and plots. [file 1471-2105-12-392-S1.PDF]

Additional file: an empirical Bayes model using a competition score for metabolite identification in gas chromatography mass spectrometry

1 Method: EM

We have two unobserved variables in the model and employ Expectation-Maximization (EM) algorithm to estimate parameters. In the E-step, we take care of latent variables through conditional expectation, resulting in target function. Then, we find the estimator which maximize the target function obtained in E-step.

1.1 E-step

In the E-step, we deal with latent (unobserved) variables through conditional expectation of complete-data log likelihood given observed data. As a result, we get target function $Q(\theta;\theta^{(k)})$. The target function is defined by:

$$Q(\theta;\theta^{(k)}) = E(l(\theta)|Z,S,\theta^{(k)}), \quad (1)$$

where $\theta = (\rho,\tau,\alpha,\beta,\phi_T,\phi_F)$ and $l(\theta) = l_1 + l_2 + l_3 + l_4$.
Note that $l_1 = \sum_{j=1}^{N}[Y_j \ln\rho + (1-\rho)\ln(1-\rho)]$,
$l_2 = \sum_{j=1}^{N}[Z_j \ln\{\lambda(\alpha;{b_j}^*)^{Y_j}\gamma(\beta;b_j)^{1-Y_j}\} + (1-Z_j)\ln\{(1-\lambda(\alpha;{b_j}^*))^{Y_j}(1-\gamma(\beta;b_j))^{1-Y_j}\}]$,
$l_3 = \sum_{j=1}^{N}\sum_{k=1}^{K_j} Y_j Z_j\{W_{jk}\ln\tau + (1-W_{jk})\ln(1-\tau)\}$, and
$l_4 = \sum_{j=1}^{N}\sum_{k=1}^{K_j}\{W_{jk}\ln f_T(S_{jk};\phi_T) + (1-W_{jk})\ln f_F(S_{jk};\phi_F)\}$
Thus, we need to calculate three different conditional expectations:
$E(Y_j|Z_j,S_{j\cdot},\theta^{(k)}) =$

$$\begin{cases} \frac{\rho\lambda\prod_{k=1}\{\tau f_T(s_{jk})+(1-\tau)f_F(s_{jk})\}}{\rho\lambda\prod_{k=1}\{\tau f_T(s_{jk})+(1-\tau)f_F(s_{jk})\}+(1-\rho)\gamma\prod_{k=1}f_F(s_{jk})} & ,\text{if } z=1 \\ \frac{\rho[1-\lambda]}{\rho[1-\lambda]+(1-\rho)[1-\gamma]} & ,\text{if } z=0 \end{cases}$$

$E(W_{jk}|Z_j,S_{jk},\theta^{(k)}) =$

$$\begin{cases} \frac{\rho\lambda\tau f_F(s_{jk})}{\rho\lambda\tau f_F(s_{jk})+[\rho(1-\tau)\lambda+(1-\rho)\gamma]f_F(s_{jk})} & ,\text{if} z=1 \\ \text{not interested} & ,\text{if } z=0 \end{cases}$$

$E(Y_jW_{jk}|Z_j,S_{jk},\theta^{(k)}) =$

$$\begin{cases} \frac{\rho\lambda\tau f_T(s_{jk})\prod_{l\neq k}\{\tau f_T(s_{jl})+(1-\tau)f_F(s_{jl})\}}{\rho\lambda\prod_{k=1}\{\tau f_T(s_{jk})+(1-\tau)f_F(s_{jk})\}+(1-\rho)\gamma\prod_{k=1}f_F(s_{jk})} & ,\text{if } z=1 \\ \text{not interested} & ,\text{if } z=0 \end{cases}$$

The formula for three conditional expectations are easily obtained with some algebra.

1.2 M-step

Here we maximize the target function given in the E-step with respect to parameters, i.e., we have to solve estimating equations for each parameter. Since α and β are coefficients in logistic regression, estimators are not existed in closed form while others have estimators in closed form. Estimators with explicit formula are represented by:

$$\hat{\rho} = \frac{1}{N}\sum_{j=1}^{N} E[Y_j|Z_j, S_j],$$

$$\hat{\tau} = \frac{1}{\sum_j \sum_k Z_j E[Y_j|Z_j]} \sum_j \sum_k Z_j E[Y_j W_{jk}|Z_j, S_j],$$

$$\hat{\mu}_{T1} = \frac{1}{\sum_i T_{1,i}} \sum_i T_{1,i} S_i,$$

$$\hat{\sigma_{T1}}^2 = \frac{1}{\sum_i T_{1,i}} \sum_i T_{1,i}(S_i - \hat{\mu}_{T1})^2$$

$$\hat{\mu}_{T2} = \frac{1}{\sum_i T_{2,i}} \sum_i T_{2,i} S_i,$$

$$\hat{\sigma_{T2}}^2 = \frac{1}{\sum_i T_{2,i}} \sum_i T_{2,i}(S_i - \hat{\mu}_{T2})^2$$

$$\hat{\mu}_{T3} = \frac{1}{\sum_i T_{3,i}} \sum_i T_{3,i} S_i,$$

$$\hat{\sigma_{T3}}^2 = \frac{1}{\sum_i T_{3,i}} \sum_i T_{3,i}(S_i - \hat{\mu}_{T3})^2$$

where $\pi_t = \frac{1}{m}\sum_i T_{t,i}$ and $T_{t,i} = \frac{\pi_t f_t(s_i)}{\sum_{j=1}^{3} \pi_j f_j(s_i)}$.
To solve nonlinear estimating equations for α and β, general-purpose optimization based on quasi Newton method is used.

1.3 Solutions of α and β

Parameter estimators for α and β are not existed in closed form and we have to solve the nonlinear estimating equations below:

Estimating equation for α
$\frac{\partial l(\theta)}{\partial \alpha_0} = \sum_{j=1}^{N} \left\{ \frac{Z_j Y_j}{\lambda} \frac{\partial}{\partial \alpha_0}\lambda - \frac{(1-Z_j)Y_j}{1-\lambda} \frac{\partial}{\partial \alpha_0}\lambda \right\} := 0$
$\frac{\partial l(\theta)}{\partial \alpha_1} = \sum_{j=1}^{N} \left\{ \frac{Z_j Y_j}{\lambda} \frac{\partial}{\partial \alpha_1}\lambda - \frac{(1-Z_j)Y_j}{1-\lambda} \frac{\partial}{\partial \alpha_1}\lambda \right\} := 0$
$\frac{\partial l(\theta)}{\partial \alpha_2} = \sum_{j=1}^{N} \left\{ \frac{Z_j Y_j}{\lambda} \frac{\partial}{\partial \alpha_2}\lambda - \frac{(1-Z_j)Y_j}{1-\lambda} \frac{\partial}{\partial \alpha_2}\lambda \right\} := 0$
Estimating equation for β

$$\frac{\partial l(\theta)}{\partial \beta_0} = \sum_{j=1}^{N} \left\{ \frac{Z_j(1-Y_j)}{\gamma} \frac{\partial}{\partial \beta_0}\gamma - \frac{(1-Z_j)(1-Y_j)}{1-\gamma} \frac{\partial}{\partial \beta_0}\gamma \right\} := 0$$
$$\frac{\partial l(\theta)}{\partial \beta_1} = \sum_{j=1}^{N} \left\{ \frac{Z_j(1-Y_j)}{\gamma} \frac{\partial}{\partial \beta_1}\gamma - \frac{(1-Z_j)(1-Y_j)}{1-\gamma} \frac{\partial}{\partial \beta_1}\gamma \right\} := 0$$
$$\frac{\partial l(\theta)}{\partial \beta_1} = \sum_{j=1}^{N} \left\{ \frac{Z_j(1-Y_j)}{\gamma} \frac{\partial}{\partial \beta_2}\gamma - \frac{(1-Z_j)(1-Y_j)}{1-\gamma} \frac{\partial}{\partial \beta_2}\gamma \right\} := 0$$

For this purpose, we employed general-purpose optimization based on quasi Newton method.

2 Some terminologies

2.1 Cosine score

The definition is

$$\vartheta = \frac{180}{\pi} cos^{-1}\left(\frac{< A, B >}{||A|| \cdot ||B||}\right), \tag{2}$$

where $< A, B >$ is inner product of spectrum A and B and $|| \cdot ||$ is the Euclidean norm. If spectra A and B have different number of peaks, then we add 0 to make both spectra have same length. For example, suppose that spectrum A has intensities $A_I = (3, 7)$ at $m/z = (3, 4)$ and spectrum B has intensities $B_I = (8, 5)$ at $m/z = (4, 5)$. Then, after adding 0, we get $A_I = (3, 7, 0)$ and $B_I = (0, 8, 5)$ at $m/z = (3, 4, 5)$.

2.2 Comparison measures

Standard comparison measures can be easily obtained by using 2×2 table (Table 1). For clarity, sensitivity, specificity, and FDR are defined as follows:

Table 1: 2×2 table: Test outcome is based on the results by any method

		Gold standard	
		Positive	Negative
Test outcome	Positive	C11	C12
	Negative	C21	C22

$$Sensitivity = \frac{C11}{C11 + C21}, \tag{3}$$

$$Specificity = \frac{C22}{C12 + C22}, \tag{4}$$

$$FDR = \frac{C12}{C11 + C12}. \tag{5}$$

In addition, ROC curve is a plot of sensitivity against 1-specificity, i.e., a mixture of sensitivity and specificity in a sense.

3 Experimental details

3.1 Experiment 1: mixture of metabolite standards

A mixture of 35 amino acids, fatty acids and organic acids were prepared in pyridine. The concentration of each acid in the mixture was 1 mg/mL. A 50 μL aliquot of the mixture was derivatized with 100 μL of N-Methyl-N-(Tert-Butyldimethylsilyl)trifluoroacetamide (MTBSTFA) for 30 min at 60^oC. All GCxGC/TOF-MS analyses were performed on a LECO Pegasus 4D time-of-flight mass spectrometer (TOF-MS) with a Gerstel MPS2 auto-sampler. The Pegasus 4D GCxGC/TOF-MS instrument was equipped with an Agilent 6890 gas chromatograph featuring a LECO two stage cryogenic modulator and secondary oven. A $30m \times 0.25mm\ id. \times 0.25\mu m$ film thickness, Rxi-5ms GC capillary column was used as the primary column for the GCxGC/TOF-MS analysis. A second GC column of $2m \times 0.10mm\ id. \times 0.10\mu m$ film thickness, BPX-50 was placed inside the secondary GC oven after the thermal modulator. The helium carrier gas flow rate was set to 1.0 mL/min at a corrected constant flow via pressure ramps. A $2\mu L$ liquid sample was injected into the liner using the splitless mode with the injection port temperature set at 260^oC. The first-dimension column oven ramp began at 60^oC with a 0.5-min hold after which the temperature was programmed to 280^oC at a rate of $8^oC/min$ and then held at this temperature for 6 min. The second-dimension column temperature was maintained 5^oC higher than the corresponding first-dimension column. The programming rate and hold times were the same for the two columns. The thermal modulator was set to $+20^oC$ relative to the primary oven and a modulation time of 5 s was used. The MS mass range was $45 - 750m/z$ with an acquisition rate of 200 spectra per second. A 700 s solvent delay was used. The ion source chamber was set at 230^oC with the MS transfer line temperature set to 260^oC and the detector voltage was $1800V$ with an electron energy of $70eV$. The LECO ChromaTOF software version 3.41 equipped with the National Institute of Standards and Technology (NIST) MS database (NIST MS Search 2.0, NIST/EPA/NIH Mass Spectral Library; NIST 2002) was used for instrument control, spectrum deconvolution and metabolite identification.

3.2 Experiment 2: rat plasma

Metabolites were extracted from a $100\mu L$ rat plasma sample using $900\mu L$ of organic solvent mixture (methanol:water = 8:1). A $50\mu L$ aliquot of plasma extract were further derivatized with N-tert-Butyldimethylsilyl-N-methyltrifluoroacetamide (MTBSTFA). The derivatized metabolite extract was spiked at a concentration of $2.5\mu g/mL$ with a deuterated six component semi-volatiles internal standard (ISTD) mixture prior to GCxGC/TOF-MS analysis by a LECO Pegasus 4D time-of-flight mass spectrometer (TOF-MS). A $30m \times 0.25mm\ id. \times 0.25\mu m$ film thickness, Rxi-5ms, GC capillary column was used as the primary column for the GCxGC/TOF-MS analysis. In the GCxGC configuration, a second column $1.2m \times 0.10mm\ id. \times 0.10\mu m$ film thickness, BPX-50, was placed inside

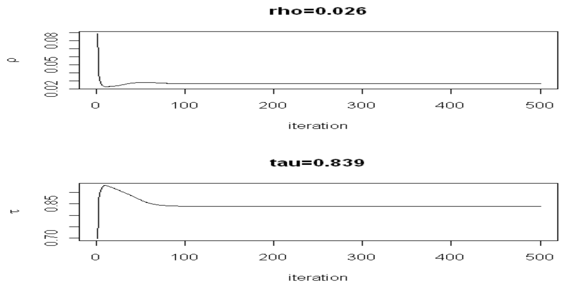

Figure 1: mixture of metabolite standards (h=30): Trace plots of ρ and τ estimate.

the LECO secondary GC oven after the thermal modulator. Helium carrier gas flow rate was set to $1.0mL$ per minute at a corrected constant flow via pressure ramps. A $1\mu L$ splitless liquid injection was made with the injection port temperature set at 260^oC. The primary column was programmed with an initial temperature of 60^oC for 0.5 minute and then ramped at 7^oC per minute to 315^oC for 8.5 minutes. The secondary column temperature program was set to an initial temperature of 65^oC for 0.5 minute and then ramped at 7^oC per minute to 320^oC with an 8.5 minutes hold time for a total runtime of 45.43 minutes. The thermal modulator was set to $+20^oC$ relative to the primary oven and a modulation time of 5 seconds was used. The MS mass range was $10-750m/z$ with an acquisition rate of 150 spectra per second. The ion source chamber was set at 230^oC with the MS transfer line temperature set to 260^oC and the detector voltage was $1800V$ with an electron energy of $-70eV$. The acquired data was processed with a user defined data processing method. The LECO ChromaTOF software version 3.41 equipped with the National Institute of Standards and Technology (NIST) MS database (NIST MS Search 2.0, NIST/EPA/NIH Mass Spectral Library; NIST 2002) was used for instrument control, spectrum deconvolution and metabolite identification.

3.3 Selection of 2000 true negatives

From the NIST MS database, we first removed some spectra which matched our sample. We then randomly selected 2000 from the rest spectra as the false spectra.

4 Results

4.1 results: mixture of metabolite standards

The trace plot of estimated proportion of the true positive metabolites $\hat{\rho}$ and estimated accuracy of our assignment $\hat{\tau}$ are given in Figure 1: Figure 2 presents

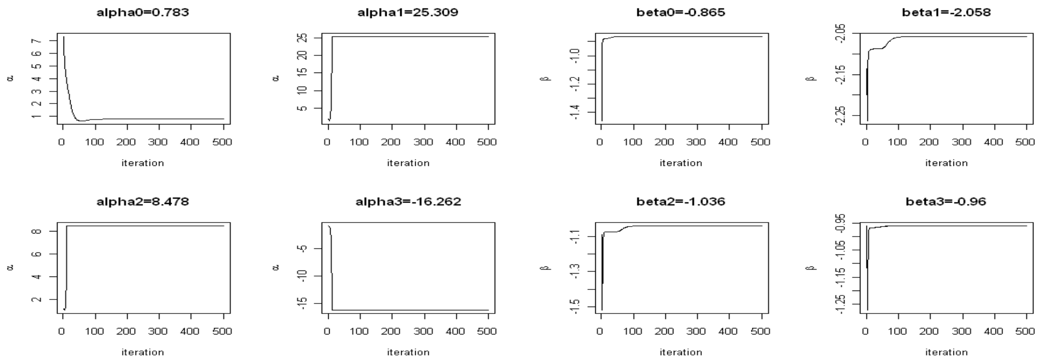

Figure 2: mixture of metabolite standards (h=30): Trace plots of α and β.

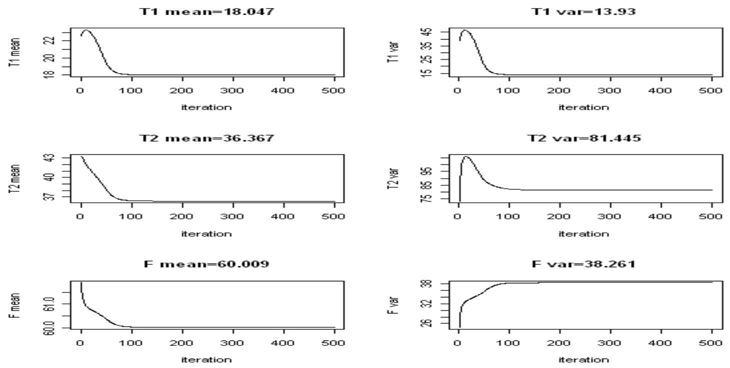

Figure 3: mixture of metabolite standards (h=30): Trace plot of parameter estimates in score functions: $(\mu_{T1}, \sigma^2{}_{T1})$, $(\mu_{T2}, \sigma^2{}_{T2})$ and $(\mu_F, \sigma^2{}_F)$.

the trace plot of parameters α. Figure 3 presents the trace plot of mean and variance of score functions.
Figure 4 presents the estimate of conditional probabilities in layer 2: functions $\hat{\lambda}$ and $\hat{\gamma}$.

To illustrate advantages of our method, we select a TP with chemical name Butanoic acid, 4-[bis(trimethylsilyl)amino]-, trimethylsilyl ester (CAS number: 39508-23-1) which is matched to 34 sample spectra. The average of the 34 similarity scores is 48.29. A half of them are covered with true score density and the rest of them are covered with false score density (Figure 5).

Some numerical information about estimation results is summarized in the Tables 2, 3 and 4.

Results for h=40 (from Figure 6 to Figure 10) are given:

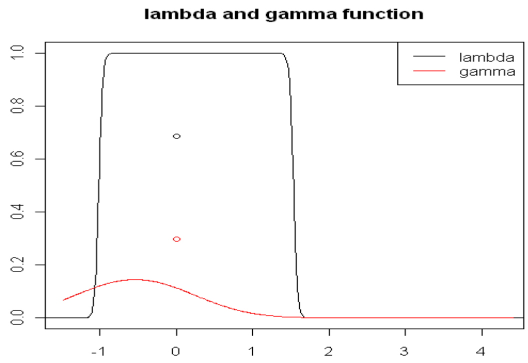

Figure 4: mixture of metabolite standards (h=30): Estimates of conditional probabilities in layer 2: functions λ and γ.

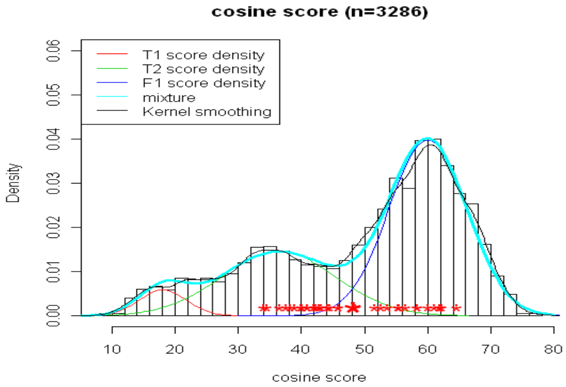

Figure 5: mixture of metabolite standards (h=30): Histogram of 34 similarity scores matched to the chemical with CAS number of 39508-23-1: big $*$ presents score average.

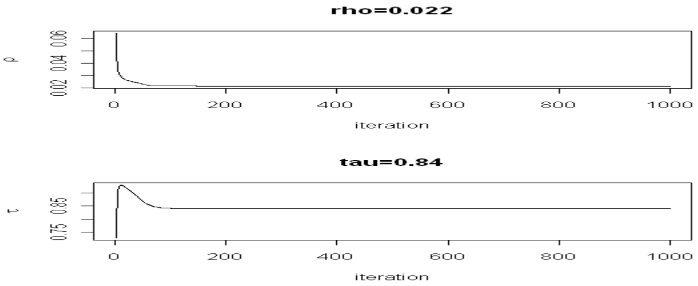

Figure 6: mixture of metabolite standards (h=40): Trace plots of ρ and τ estimate.

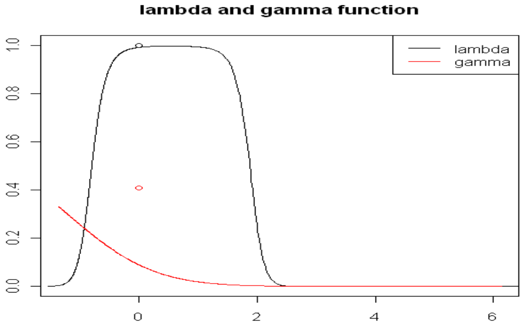

Figure 7: mixture of metabolite standards (h=40): Estimates of conditional probabilities in layer 2: functions λ and γ.

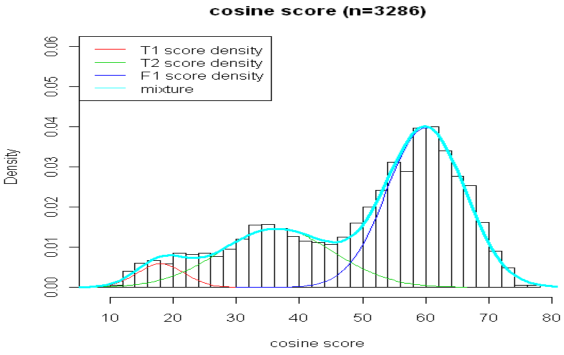

Figure 8: mixture of metabolite standards (h=40): Three estimated score functions and mixture score function.

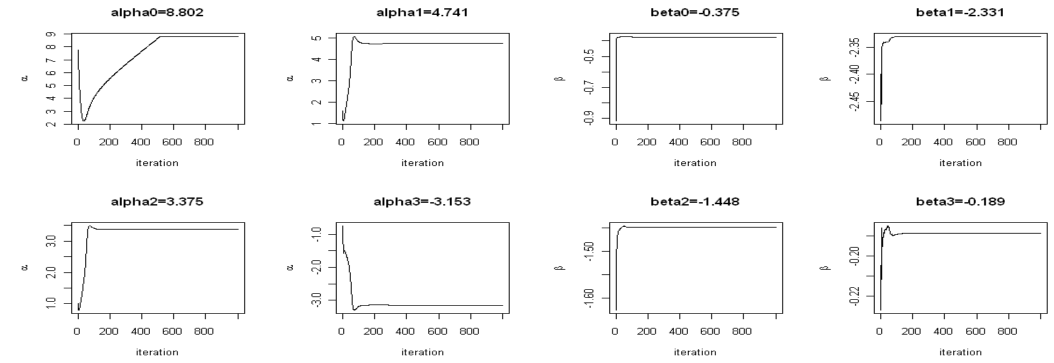

Figure 9: mixture of metabolite standards (h=40): Trace plots of α and β.

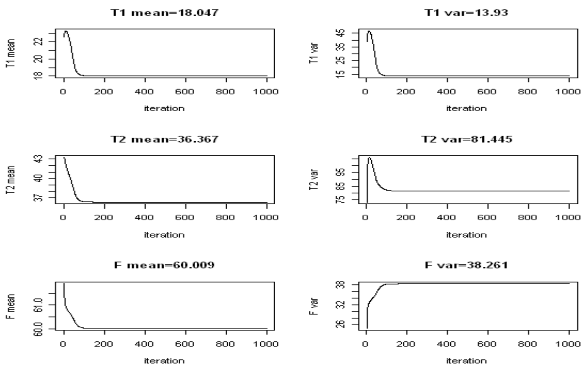

Figure 10: mixture of metabolite standards (h=40): Trace plot of parameter estimates in score functions: $(\mu_{T1}, \sigma^2{}_{T1})$, $(\mu_{T2}, \sigma^2{}_{T2})$ and $(\mu_F, \sigma^2{}_F)$.

Table 2: Summary of results from HBM (cutoff=30): mixture of metabolite standards

cutoff=0.087	TP	TN	cutoff=0.174	TP	TN
EP	32	6	EP	32	2
EN	20	1994	EN	20	1998
cutoff=0.342	TP	TN	cutoff=0.5	TP	TN
EP	31	1	EP	31	1
EN	21	1999	EN	21	1999
cutoff=0.574	TP	TN	cutoff=0.643	TP	TN
EP	31	0	EP	31	0
EN	21	2000	EN	21	2000
cutoff=0.766	TP	TN	cutoff=0.819	TP	TN
EP	30	0	EP	30	0
EN	22	2000	EN	22	2000
cutoff=0.866	TP	TN	cutoff=0.906	TP	TN
EP	30	0	EP	30	0
EN	22	2000	EN	22	2000
cutoff=0.94	TP	TN	cutoff=0.966	TP	TN
EP	29	0	EP	29	0
EN	23	2000	EN	23	2000

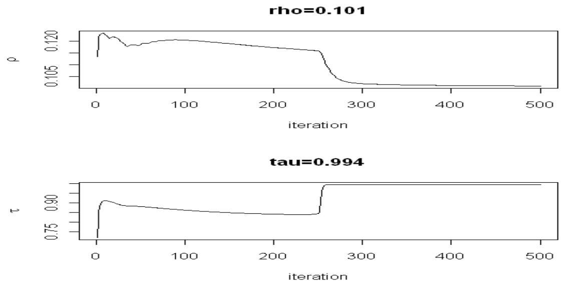

Figure 11: Rat plasma: Trace plots of ρ and τ estimate.

4.2 results: rat plasma

The trace plot of estimated proportion of the true positive metabolites $\hat{\rho}$ and estimated accuracy of our assignment $\hat{\tau}$ are given in Figure 11: Figure 12 presents the trace plot of parameters α. Figure 13 presents the trace plot of mean and variance of score functions.
Based on the estimates, $\hat{\rho}$ and $\hat{\tau}$, we can say that about 10.1% (i.e. 354) of library spectra are present in our sample and that our identification is correct with the probability of 0.99. Figure 14 presents the estimate of conditional

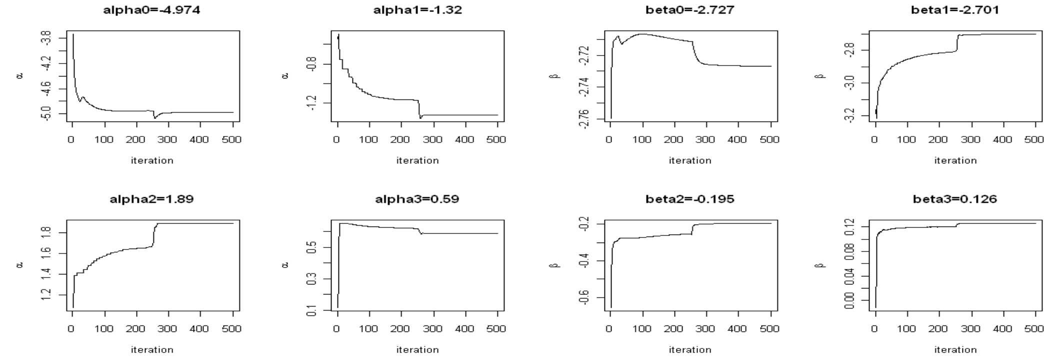

Figure 12: Rat plasma: Trace plots of α and β.

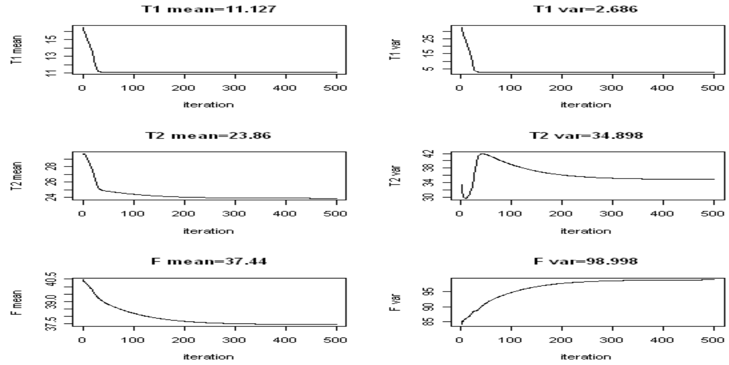

Figure 13: Rat plasma: Trace plot of parameter estimates in score functions: $(\mu_{T1}, {\sigma^2}_{T1})$, $(\mu_{T2}, {\sigma^2}_{T2})$ and $(\mu_F, {\sigma^2}_F)$.

Table 3: Summary of results from HBM (cutoff=40): mixture of metabolite standards

cutoff=0.087	TP	TN	cutoff=0.174	TP	TN
EP	32	4	EP	32	2
EN	20	1996	EN	20	1998
cutoff=0.342	TP	TN	cutoff=0.5	TP	TN
EP	31	1	EP	31	1
EN	21	1999	EN	21	1999
cutoff=0.574	TP	TN	cutoff=0.643	TP	TN
EP	31	1	EP	30	0
EN	21	1999	EN	22	2000
cutoff=0.766	TP	TN	cutoff=0.819	TP	TN
EP	30	0	EP	30	0
EN	22	2000	EN	22	2000
cutoff=0.866	TP	TN	cutoff=0.906	TP	TN
EP	30	0	EP	30	0
EN	22	2000	EN	22	2000
cutoff=0.94	TP	TN	cutoff=0.966	TP	TN
EP	30	0	EP	29	0
EN	22	2000	EN	23	2000

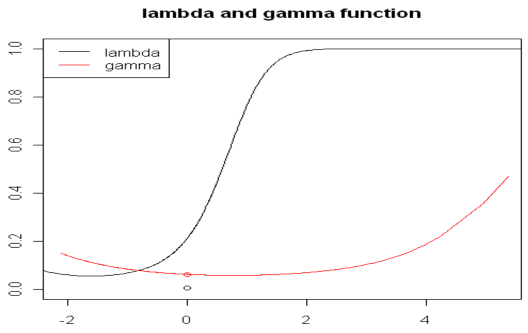

Figure 14: Rat plasma: Estimates of conditional probabilities in layer 2: functions λ and γ.

probabilities in layer 2: functions $\hat{\lambda}$ and $\hat{\gamma}$. Since λ is interpreted as the likelihood that a spectra in library is matched to some sample spectra given that it is in sample, our method provides us with very high likelihood of matching over the wide range of b_j. Also, we can say that such match is correct with high confidence. Three estimated score curves and mixture score function are given

Table 4: Summary of results from Naive: mixture of metabolite standards

cutoff=0.087	TP	TN	cutoff=0.174	TP	TN
EP	43	374	EP	43	374
EN	9	1626	EN	9	1626
cutoff=0.342	TP	TN	cutoff=0.5	TP	TN
EP	43	344	EP	41	92
EN	9	1656	EN	11	1908
cutoff=0.574	TP	TN	cutoff=0.643	TP	TN
EP	35	14	EP	27	2
EN	17	1986	EN	25	1998
cutoff=0.766	TP	TN	cutoff=0.819	TP	TN
EP	14	0	EP	8	0
EN	38	2000	EN	44	2000
cutoff=0.866	TP	TN	cutoff=0.906	TP	TN
EP	6	0	EP	3	0
EN	46	2000	EN	49	2000
cutoff=0.94	TP	TN	cutoff=0.966	TP	TN
EP	1	0	EP	0	0
EN	51	2000	EN	52	2000

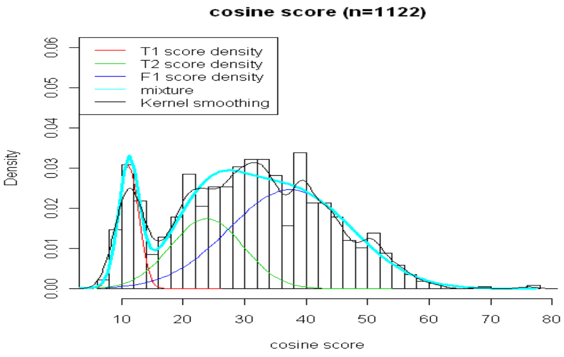

Figure 15: Rat plasma: Histogram of similarity scores and three estimated score functions and mixture score function.

in Figure 16:

4.3 Goodness of fit test

In this section, we did goodness of fit (GOF) test to check how well our density estimate fits the data. To do that, we modified Kolmogorov-Smirnov (KS) GOF and Anderson-Darling (AD) GOF test because both methods are not available

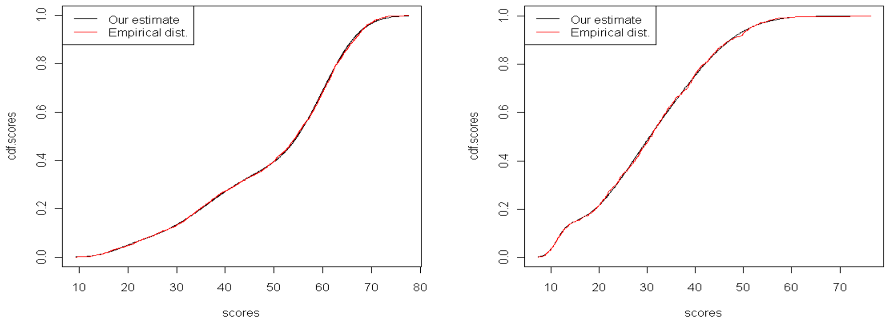

Figure 16: Left: Two CDF for standard mixture data. Right: two CDF for rat plasma data

for gaussian mixture. Furthermore, since there is no critical value for the test of null hypothesis that the data follow the specified distribution, here, gaussian mixture, we cannot conclude the significance of hypothesis statistically. Thus, we provide two cumulative distribution function: one from the data and the other from our estimated mixture distribution. Based on CDF plots (Figure 16), we say that the data follow the estimated distribution empirically. Moreover, we got test statistic (KS=0.0113 and AD=0.7996) for standard mixture data and test statistic (KS=0.0146 and AD=0.2433) for rat plasma data. As mentioned, since the critical values for gaussian mixture are not available and we cannot calculate exact p-value here. However, if we compare test statistics to critical value for normal distribution which is available, we cannot reject null hypothesis at the level of 0.01, i.e., we can say that the data follow the gaussian mixture. Notice that for reference we used critical values for normal distribution, not those for gaussian mixture. Through the comparison of test statistic with critical value for normal distribution, it seems that the data follow the estimated distribution empirically.

4.4 Comparison

For comparison, other than naive method, we implemented three other methods: the NIST MS dot product, the weighted dot product, and composite similarity based on the methods developed by Stein and Scott (1994). The detailed definition can be found in Stein and Scott (1994). We examined the performance for each method in terms of sensitivity, 1-specificity, and FDR. For given specificity values, corresponding ROC curves and FDR plots are given in Figure 17. Based on the plots, the proposed method prevails over others in terms of the sensitivity and FDR. The second best is naive method. Furthermore, we also provide the tables (Table 5 to Table 7) including all numerical values such as sensitivity, specificity and FDR.

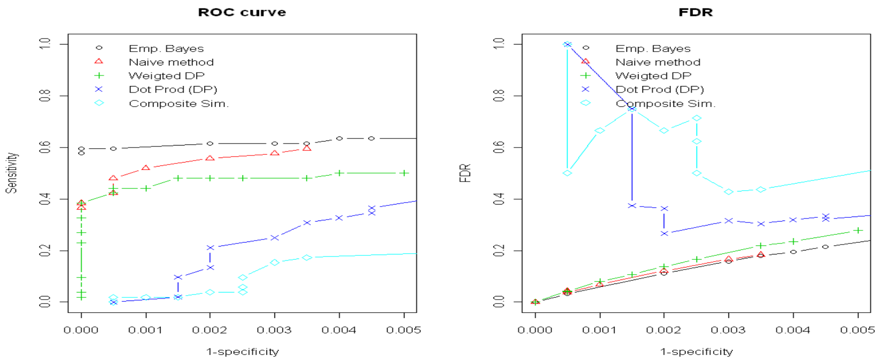

Figure 17: ROC curve and FDR plot against 1-specificity for each method

Table 5: Dot product: sensitivity, specificity and the false discovery rate (FDR).

cutoff	Sensitivity	1-Specificity	FDR	cutoff	Sensitivity	1-Specificity	FDR
0.7990	0.5192	0.0060	0.3077	0.8069	0.5000	0.0060	0.3158
0.8149	0.5000	0.0060	0.3158	0.8229	0.5000	0.0060	0.3158
0.8308	0.5000	0.0060	0.3158	0.8388	0.4808	0.0060	0.3243
0.8467	0.4423	0.0060	0.3429	0.8547	0.4423	0.0060	0.3429
0.8627	0.4423	0.0060	0.3429	0.8706	0.4038	0.0060	0.3636
0.8786	0.4038	0.0060	0.3636	0.8865	0.4038	0.0055	0.3438
0.8945	0.3654	0.0045	0.3214	0.9024	0.3654	0.0045	0.3214
0.9104	0.3654	0.0045	0.3214	0.9184	0.3462	0.0045	0.3333
0.9263	0.3269	0.0040	0.3200	0.9343	0.3077	0.0035	0.3043
0.9422	0.2500	0.0030	0.3158	0.9502	0.2115	0.0020	0.2667
0.9582	0.1346	0.0020	0.3636	0.9661	0.0962	0.0015	0.3750
0.9741	0.0192	0.0015	0.7500	0.9820	0.0000	0.0005	1.0000

It would be noteworthy that the probability score in the MS search is different from the PBM library search, which was introduced by Atwater et al. (1985), to our knowledge (Stein, 1994).

Table 6: Weighted dot product: sensitivity, specificity and the false discovery rate (FDR).

cutoff	Sensitivity	1-Specificity	FDR	cutoff	Sensitivity	1-Specificity	FDR
0.7910	0.5000	0.0050	0.2778	0.7990	0.5000	0.0050	0.2778
0.8069	0.5000	0.0040	0.2353	0.8149	0.4808	0.0035	0.2188
0.8229	0.4808	0.0025	0.1667	0.8308	0.4808	0.0020	0.1379
0.8388	0.4808	0.0020	0.1379	0.8467	0.4808	0.0020	0.1379
0.8547	0.4808	0.0015	0.1071	0.8627	0.4423	0.0010	0.0800
0.8706	0.4423	0.0005	0.0417	0.8786	0.4423	0.0005	0.0417
0.8865	0.4231	0.0005	0.0435	0.8945	0.3846	0.0000	0.0000
0.9024	0.3846	0.0000	0.0000	0.9104	0.3269	0.0000	0.0000
0.9184	0.2692	0.0000	0.0000	0.9263	0.2308	0.0000	0.0000
0.9343	0.0962	0.0000	0.0000	0.9422	0.0962	0.0000	0.0000
0.9502	0.0385	0.0000	0.0000	0.9582	0.0192	0.0000	0.0000
0.9661	0.0192	0.0000	0.0000				

Table 7: Composite similarity: sensitivity, specificity and the false discovery rate (FDR).

cutoff	Sensitivity	1-Specificity	FDR	cutoff	Sensitivity	1-Specificity	FDR
0.7114	0.1923	0.0055	0.5238	0.7194	0.1731	0.0035	0.4375
0.7273	0.1538	0.0030	0.4286	0.7353	0.0962	0.0025	0.5000
0.7433	0.0577	0.0025	0.6250	0.7512	0.0385	0.0025	0.7143
0.7592	0.0385	0.0020	0.6667	0.7671	0.0385	0.0020	0.6667
0.7751	0.0192	0.0015	0.7500	0.7831	0.0192	0.0010	0.6667
0.7910	0.0192	0.0005	0.5000	0.7990	0.0192	0.0005	0.5000
0.8069	0.0192	0.0005	0.5000	0.8149	0.0192	0.0005	0.5000
0.8229	0.0000	0.0005	1.0000	0.8308	0.0000	0.0005	1.0000
0.8388	0.0000	0.0005	1.0000				
